# Advances in population-based interventions to control falciparum malaria

**DOI:** 10.1093/trstmh/traf088

**Published:** 2025-09-18

**Authors:** Samuel E Glossop, Thomas J Peto, Bipin Adhikari

**Affiliations:** School of Medicine and Biomedical Sciences, University of Oxford, Oxford OX3 9DU, UK; Mahidol Oxford Tropical Medicine Research Unit, Faculty of Tropical Medicine, Mahidol University, Bangkok 10400, Thailand; Centre for Tropical Medicine and Global Health, Nuffield Department of Clinical Medicine, University of Oxford, Oxford OX3 7LG, UK; Mahidol Oxford Tropical Medicine Research Unit, Faculty of Tropical Medicine, Mahidol University, Bangkok 10400, Thailand; Centre for Tropical Medicine and Global Health, Nuffield Department of Clinical Medicine, University of Oxford, Oxford OX3 7LG, UK

## Abstract

Malaria is a complex disease and transmission can be prevented in multiple ways. A range of interventions to achieve this became widely available from the year 2000, and cases continually declined, but progress has plateaued since 2015. This review aims to cover the population-level prevention strategies responsible for this and those that could continue this progress, focussing on how they can be successfully integrated. Insecticide-treated nets (ITNs) made the most substantial contribution to reducing malaria mortality, but their distribution, access and use remains suboptimal while development of insecticide resistance requires continuous adaptation. Chemoprevention provides protections to tens of millions of people, primarily children in sub-Saharan Africa, but is also threatened by the emergence and spread of drug resistance. These strategies may have reached a point of saturation for reducing morbidity and mortality, thus calling for innovative developments to build upon more basic approaches such as accurate early diagnosis, appropriate treatment and improved housing. The R21/Matrix-M vaccine is a significant improvement over the RTS,S/AS01 vaccine, with greater efficacy, lower cost and scalable mass production. Field trials of current monoclonal antibodies (mAbs) suggest that next-generation mAbs could be a promising tool for seasonal chemoprophylaxis. Furthermore, gene drives may have the potential to eradicate entire populations of malaria-transmitting mosquitoes. A multifaceted approach combining these new strategies with traditional approaches (ITNs and chemoprevention) offers a framework to reinvigorate progress towards malaria elimination.

## Introduction

In 2023 there were 263 million cases of malaria, resulting in an estimated 597 000 deaths. Most of the burden was caused by *Plasmodium falciparum* and was borne by children <5 y of age in Africa (76% of all deaths).^1^ Between 2000 and 2015 there was a significant reduction in malaria burden, central to which was prevention measures such as insecticide-treated nets (ITNs).^2^ This reduction has since plateaued, but subsequent developments have rekindled optimism, with leaders from the 11 highest-burden countries signing the Yaoundé Declaration, pledging to accelerate progress towards elimination. Furthermore, the commitment of all World Health Organization (WHO) member states remains enshrined in the Global Technical Strategy (GTS). We focus here on population tools for the prevention of *P. falciparum* malaria in the highest burden group, children <5 y of age in the highest transmission regions, who are the primary target for interventions such as seasonal malaria chemoprevention (SMC) and vaccines. The strategies might also be appropriate for lower transmission regions and lower risk groups, but challenges such as the limited supply of vaccines, resistance concerns, declining cost-effectiveness with lower incidence and greater proportion of *Plasmodium vivax* must also be considered.

First, we discuss current trends in two well-established measures, ITNs/long-lasting insecticide-treated nets (LLINs) and SMC. We then cover novel developments, the WHO approval of the RTS,S/AS01 and R21/Matrix-M malaria vaccines, recent developments in monoclonal antibodies (mAbs) and the potential of gene drive mosquitoes. We aim to build on a recent commentary^3^ by providing a detailed discussion of the technical considerations, limitations and evidence base for the malaria prevention and control measures that underpin malaria elimination efforts. We then outline the current status of and future directions for each intervention, considering how these strategies can be targeted and unified into a coherent policy. This is summarized in Table 1.

**Table 1.** The challenges facing and future directions for malaria control.

Control method	Challenges	Future directions	Current status
ITNs	Access to nets^7–9^ Utilization of owned nets^10^ Developing physiological and behavioural resistance^13,14,16–19^	Improved distribution/allocation^7^ Development of more comfortable and durable nets Designs intended for specific groups^10^ Development of new insecticides Community engagement^11^	3 billion nets supplied over the last 20 y. 73% of households in sub-Saharan Africa own a net, 59% of children <5 y of age sleep under one. Distribution campaigns (often free) continue.^1^
SMC	Parasite resistance^26–28,31^ Potential rebound malaria	New drugs^29,30^ Expansion of coverage to children ages 5–10 y and into less seasonal areas^22,24,25^	Offered to children <5 y of age in 19 countries in sub-Saharan Africa, primarily in the Sahel.^20^
Vaccines	Long-term efficacy^33,34,38,40^ Cost^38^ Number of doses required^33,38^ Compliance^32^	Cost reductions and fewer doses^33,35^ Combination of vaccines (or polyvalent vaccines)^42^ Transmission-blocking vaccines^41^	R21/Matrix-M has completed phase III trials. Mass administration has begun and continues to be upscaled.^38^
mAbs	Cost^21,47^ Administration^44,45^	Higher potency^48,49^ Lower production cost^48^ Subcutaneous delivery^45^	Phase II trials completed with further promising candidates in development.^46,48^
Gene drives	Ethical concerns^57,58^	Field trials with ethical considerations^57,58^	Technical issues resolved, awaiting decisions on how to test/implement.^50^
Funding	Declining Insecure	Increasing the number of funders Securing longer-term commitments	Funding can vary significantly each year and is declining overall.^1^
Elimination and eradication	Uncertain funding Limited efficacy Resistance	Investment into continued research investigating how to mitigate resistance, improve efficacy and combine different strategies^36^	Progress is inconsistent across countries. Commitments are enshrined in the WHO GTS and more recently the Yaoundé Declaration.^1^

## ITNs

For >20 y, LLINs have been the cornerstone of malaria control and a WHO core intervention.^4^ They have been shown to reduce all-cause mortality in children by 17%,^5^ placental malaria by 21% and low birth weight by 23%.^6^ Furthermore, a modelling study suggests they were responsible for 68% of the decline in malaria between 2000 and 2015.^2^

Campaigns of LLIN mass distribution to entire populations have been established, aiming to achieve coverage >80%. However, this has been largely unsuccessful and decreasing funding for global health is likely to further undermine this effort. LLIN distribution campaigns typically occur every 3 y, at rates of 1.8 people per net, despite modelling suggesting median retention times of 1.64 y^7^ and that allocation efficiency declines at high coverage.^8^ Continuous delivery strategies could provide more efficient coverage; limits on the number of nets per household may also need to be removed,^9^ as they disadvantage large families.

Ownership of LLINs does not guarantee utilisation; such non-use is most commonly due to the discomfort caused by heat and a perceived low mosquito density.^10^ Health education interventions are one way of combating bed net fatigue and have been found to have significant impacts on use.^11^ Changes to net fibres could further improve adherence and also durability, making nets more cost-effective. Use is also limited in population groups where current house designs encounter intractable issues, such as the eve gaps of traditional houses (e.g. mud huts and stilt houses), or for farmers and travellers who rarely have access to, or the ability to use, their ITNs when sleeping away from home (e.g. overnight stays during farm and forest work). One potential solution to this is a portable insecticide-treated hammock, but their effectiveness is limited by adherence and lack of protection during non-sleeping hours, among other practical issues.^10^ Most recently, housing modifications and novel designs have been proposed to protect from mosquito bites, and thus malaria.^12^

Besides coverage and usage problems, ITNs are becoming less effective as mosquitoes increasingly bite during the daytime and outdoors, evading net protection.^13^ Specific species also pose new challenges, *Anopheles dirus* and *Anopheles minimus* tend to bite at dusk, a common time for outdoor meals and recreation, while the invasive *Anopheles stephensi* thrives in urban environments and resists current insecticides.^14^ This necessitates additional protection before bedtime, but solutions such as topical and spatial repellents have yet to demonstrate effective protection during this period.^15^

The effectiveness of the insecticide component is threatened by the spread of physiological insecticide resistance, which develops faster than behavioural resistance,^16^ suggesting research should prioritise new insecticides for incorporation into dual active ingredient ITNs (DAI-ITNs). For example, the addition of chlorfenapyr (an insecticide with a different mechanism of action) to pyrethroid ITNs led to a 45% reduction in malaria.^17^ Such DAI-ITNs were strongly recommended by the WHO in March 2023.^1^ Piperonyl butoxide (PBO) has also been trialled and received conditional recommendation, but is already encountering vector resistance amidst suggestions it was upscaled a decade late^18^ (PBO is a synergist that inhibits the enzymes mediating pyrethroid resistance, reducing resistance to both). A direct comparison of the two found a 32% reduction with chlorfenapyr–pyrethroid in comparison to PBO–pyrethroid,^19^ but having multiple viable options will aid ongoing control efforts based on local resistance profiles.

In summary, the effectiveness of ITNs is primarily limited by access, suggesting improved distribution programs will be of the greatest benefit.^7^ Effectiveness also depends on behavioural adherence, net lifespan and vector resistance. New developments in ITNs should focus on all these challenges and potential integration with other prevention strategies.

## Chemoprevention

SMC is the intermittent administration of a curative dose of antimalarial medicine in seasonal transmission areas, regardless of infection status. Since 2012, using up to four monthly doses of sulfadoxine-pyrimethamine and amodiaquine (SP-AQ) has been recommended by the WHO for children <5 y of age, with the most recent meta-analysis showing a 73% reduction in uncomplicated malaria during the high transmission season.^20^ The high effectiveness and low cost (US$3.63 per child per year) compared with the average price of treating malaria infections makes SMC cost-saving due to the large number of malaria infections it prevents.^21^

Given this success, the expansion of SMC has been considered. Updated WHO guidelines include more countries and permit them to determine the number of cycles offered, allowing countries with 5- or 6-month transmission seasons, such as Ghana, to implement SMC nationwide after it showed a protective effectiveness of 38%.^22^ However, increasing the number of cycles may not be feasible if it interferes with recipient's routine work, such as farming, and might also cause suboptimal dosing and poor coverage, contributing to resistance and ultimately reduced effectiveness.^23^ This research also supports concerns that changing climate patterns and altered seasonality might limit the success of SMC. Expanding SMC to children 5–10 y of age has been trialled in Senegal, demonstrating a 60% reduction in cases.^24^ A subsequent study that reported outcomes by age group showed SMC had a similar effect in children ages 5–9 y^25^ and also led to a 26% reduction in older, untreated groups. The number of cycles, ages covered and the feasibility of each continues to be researched given the significant potential to reduce the malaria burden.

For any antimalarial, there is the risk that drug resistance will develop, especially when used at the scale of SMC. However, this has thus far progressed slowly. One study found resistance variants in only 0.05% of parasites after 2 y of SMC use,^26^ while another found no increase in most resistance markers during 3 y of SMC implementation. However, one marker rose significantly and deep sequencing showed an increase in two further resistance mutations.^27^ This slow and uneven development of resistance allows SMC to remain effective—the effectiveness estimated by meta-analyses throughout the past decade has remained constant.^20^ Furthermore, a recent study in Burkina-Faso found malaria in SMC-eligible children was more likely a result of low drug levels caused by missed cycles than resistance, with a <1% prevalence of mutations predicting high-level SP resistance.^28^ Regardless, development of new, alternative drugs would be welcome.

Dihydroartemisinin-piperaquine (DHA-PQ) has been proposed as an alternative, as it has similar efficacy and even fewer side effects,^29^ but this is controversial due to its use as a first-line treatment for symptomatic malaria,^20,30^ especially given the emergence of resistance to artemisinin combination therapies in different parts of Africa.^1^ Furthermore, in South Sudan, an area with presumed SP resistance, SMC has remained effective, with a 53% reduction in uncomplicated malaria observed.^31^

Chemoprevention can also be delivered during scheduled healthcare contacts. Pregnant women receive intermittent preventive treatment in pregnancy during routine antenatal visits. This is associated with a 20% reduction in placental or maternal malaria at delivery.^4^ Perennial malaria chemoprevention was previously known as intermittent preventive treatment in infants but was recently renamed to reflect the recommendation that children up to 2 y of age should be covered. It uses the childhood vaccination schedule for delivery of SP to children <2 y of age; it is used in areas where SMC is unsuitable (i.e. in the absence of significant seasonality) and has achieved a lower effectiveness (30%).^4^ Alternative/additional drugs to SP should therefore be considered, as should strategies for improving coverage and timing of doses. It may be necessary to combine chemoprevention with vaccination, which might also mitigate evolving resistance.

## Vaccines

In 2022, the RTS,S/AS01 malaria vaccine was prequalified by the WHO and was soon followed by the 2023 approval of the R21/Matrix-M vaccine, developed with a focus on low-cost mass production. Thus far, vaccine acceptance has been high, but this varies between settings and should be continually monitored, with strategies adjusted accordingly.^32^

Initial results for the RTS,S/AS01 vaccine were promising; vaccine efficacy against clinical malaria at 14 months was 50.4% in children whose initial age was 5–17 months given three doses. However, overall efficacy declined to 36.3% over 4 y, and even with a booster at 20 months, fell to just 12.3% between months 33 and 48.^33^ Furthermore, one study with a 7-year follow-up demonstrated rebound malaria in children with higher-than-average exposure 5 y post-vaccination. As a result, overall efficacy waned to only 4.4%.^34^ The rebound effect is discussed later.

Given this low efficacy, varied schedules and fractional dosing have been trialled^35^ and although they do not improve efficacy, they allow flexibility of delivery and improved cost-effectiveness and coverage given the limited vaccine supply. Incorporating the RTS,S/AS01 vaccine into the expanded program on immunisation (giving the first dose at 6–12 weeks) reduced 3 y efficacy to 25.9%;^33^ it is unclear whether potential benefits such as improved compliance and reduced delivery costs will offset this. Trials now accept that annual boosters of RTS,S/AS01 will be necessary; a large-scale study found SMC and seasonal RTS,S/AS01 vaccination provided equal protection against clinical malaria; no significant difference was seen in hazard ratios (HRs; 0.92 [95% confidence interval {CI} 0.84 to 1.01]) for RTS,S/AS01 compared with SMC. Moreover, SMC+RTS,S/AS01 had an additive protective efficacy of 62.8% when compared with SMC alone.^36^

Although it is unlikely to be used to the extent of R21/Matrix-M, the challenges faced by RTS,S/AS01 will be informative in the rollout of its successor. Like RTS,S/AS01, R21/Matrix-M targets circumsporozoite protein, but achieves a higher surface concentration on virus-like particles.^37^ Phase III trials have confirmed the vaccine shows high efficacy over the first year (75% at seasonal sites and 68% at standard sites),^38^ meeting the GTS target.^39^ Most importantly, R21/Matrix-M can be produced at the scale needed at an affordable price.

Like RTS,S/AS01, albeit to a lesser extent, R21/Matrix-M efficacy declines, falling from 80% in months 1–3 to 67% in months 10–12 at seasonal sites (79% to 63% for standard sites).^38^ Currently, only phase II trials have been ongoing long enough to investigate long-term efficacy. They have shown 80% efficacy in the year after a booster at month 14.^40^ These preliminary results are promising and suggest R21/Matrix-M efficacy is more sustained than RTS,S/AS01.

Rather than preventing infection, transmission blocking vaccines target parasites in the mosquito vector, thereby preventing vaccinated individuals from transmitting malaria and impeding community spread; they are likely to be key to elimination programs. The most researched antigen targets, Pfs25 and Pfs230, are found in the zygote and gamete stages, respectively. In phase I trials, Pfs230D1-EPA/Alhydrogel showed persistent transmission-reducing activity (73.7% after 10 weeks), while Pfs25-EPA/Alhydrogel did not (58.2% after the fourth dose).^41^ Preclinical models have shown that a co-formulation of Pfs25-IMX313 with RTS,S/AS01 does not reduce the immunogenicity or antibody response to either component,^42^ offering the potential to significantly improve vaccination efforts by combining vaccines.^40^

For a broader view of the vaccines in development see Duffy et al.^43^

## mAbs

It has been known for >60 y that gamma globulin transfused from immune adults reduces parasitaemia, but significant investment into antimalaria mAbs (proteins that bind to specific antigens, typically the circumsporozoite protein on *P. falciparum*, inactivating it) only began in 2018.^1^ This investment has returned promising candidates with key features such as a long half-life, easy administration, high potency and low production costs.

In 2020, the first antimalaria mAb, CIS43LS, was trialled, demonstrating safety and efficacy.^44^ Unfortunately, the impracticality of intravenous administration and the cost of doses makes CIS43LS unsuitable for low- and middle-income countries, but it was nonetheless an exciting proof of concept.

Next-generation mAbs such as LSL9 were found to be three times more potent than CIS43LS in preclinical models^45^ and when delivered subcutaneously in 150-mg doses were 67% efficacious over 6 months.^46^ Production costs are estimated to be US$50/g, giving per-cycle drug costs of $3 per child (similar to SMC^21^). In the future, production costs may decrease to $20/g^47^ and potency will increase, making a given effectiveness cheaper. One promising candidate for achieving this is MAM01, designed with a focus on cost and scalability determinants such as route of administration and stability.^48^ In unpublished work, MAM01 has been shown to be equipotent to LSL9^49^ and is undergoing phase I trials (NCT05891236). To progress further and become a significant pillar of control programs, prices must continue to fall and phase III trials will need to validate safety and efficacy.

## Gene drives

Like mAbs, gene drives have only recently been applied to malaria control, facilitated by a growing understanding of CRISPR-Cas9. In a gene drive, heterozygotes are converted to homozygotes, increasing the inheritance of the gene(s). The effectors associated with the drive construct classify it as either population modifying (conferring a resistance phenotype to the vector) or suppressing (reducing the vector population's reproductive capacity). Gene drives are predicted to remain stable in populations for a period sufficient to consolidate progress made and support a rapid drive towards elimination.^50^

Population suppression disrupts genes, causing female sterility. First-generation gene drives achieved inheritance rates >95% but struggled with imposed fitness costs and subsequent resistance, a problem solved by targeting the highly conserved gene *doublesex*.^51^ This led to 100% introduction and population collapse within 7–11 generations, a finding replicated in large cage trials^52^ (the most realistic model trialled to date). Encouragingly, this strategy can be combined with sex-distorting drives to accelerate population collapse and reduce resistance risks.^53^ Alternatively, population modification has shown spread at a similar speed, good efficacy (predicted to reduce vectorial capacity 65–90%) and the ability to offset fitness costs.^54^ Rather than vector resistance (there is no pressure on the mosquitoes), parasite resistance is the key challenge, arising in response to the rapid introduction of a strong selection pressure. Here, CopyCat elements might be useful. These are systems propagated by the Cas9 provided by the initial drive to ‘update’ effector genes and counteract resistance.^55^ The Cas9 can also be used by neutralising elements, offering the possibility of reversing a drive.^56^

Given these successes, it has been argued the technical barriers have been resolved.^50^ Nonetheless, implementation faces ethical challenges, as unlike the other strategies, participation is not an individual choice with predictable consequences. The challenges faced can be broadly categorised as: what are the alternatives?; what is the role of humans in nature?; and how should the uncertainty regarding the effects of gene drives be approached?^57^ For example, will the phase II field testing described by the WHO prove successful and validate the ‘causal pathway’?^58^ More importantly, which stakeholders should make these decisions, given their potential global impact?

## Challenges

### Rebound malaria

A common concern with malaria prevention is the negative effect it could have on the acquisition of natural immunity, which requires repeated exposure to malaria. The potential consequence is ‘rebound malaria’, which can be categorised as ‘delayed’ or ‘resurgent’.^59^

In delayed malaria, the withdrawal of an intervention at a prespecified age increases the risk of malaria above what it would have been if the intervention had not initially been given.^59^ However, follow-up 6 y after a program to improve ITN use showed that as well as the initial period of success (22% reduction in all-cause mortality), mortality did not differ 6 y later (HR 1.01 [95% CI 0.86 to 1.19]).^60^ Similarly, the incidence rate ratio (IRR) in the post-intervention transmission season was 1.87 vs 1.73 for the intervention and placebo groups of an SMC trial (IRR 1.09 [CI 0.99 to 1.21]).^61^ Taken together, this suggests delayed malaria is minimal and smaller than the original protection. Additionally, more recent studies with RTS,S/AS01 show that even though efficacy may be negative at later time points, overall efficacy is still positive^35^ or at least neutral.^34^ This effect should therefore be considered when analysing efficacy and highlights the importance of follow-up, but so far the negative effects of rebound malaria have always been smaller than the benefit gained.

Sustained reductions in malaria will reduce the health incentives of control measures and their cost-effectiveness, potentially affecting adherence and funding, respectively. If interventions are withdrawn or coverage declines before a reduction in parasite prevalence, this might lead to resurgent malaria,^59^ where malaria burden returns to pre-intervention levels. ITNs are likely to be least susceptible to this, as they prevent bites, making their use dependent on mosquitoes rather than malaria. This highlights how successive interventions require less frequent cooperation; ITNs require a nightly choice to use them, while vaccines, SMC and mAbs require cooperation only a few times per year. Nonetheless, all still require sustained cooperation as their effectiveness declines.

### Resistance

All interventions exert a selection pressure, driving resistance against them and reducing their effectiveness. For ITNs, the pressure is exerted on the mosquito, which develops resistance against insecticides, while for SMC, vaccines and mAbs, the parasite develops resistance. Targeted use of control measures (e.g. only children <5 y of age in seasonal areas) will minimise the pressure, selection and resulting resistance, while ubiquity, such as for ITNs, will accelerate the emergence and spread of resistance. Fortunately, their action as a physical barrier will mitigate losses in insecticidal properties. Finally, the consequences of developing resistance to the parasite/vector should also be considered, as was the case with targeting *doublesex*, a highly conserved target. The simplest way to avoid the risks associated with accumulating resistance and safeguard the progress made is a drive towards elimination within the next decade.

### Combination strategies

For >20 y, ITNs have offered substantial protection for vulnerable populations, in part due to the feasibility of periodic distribution to large numbers of people. Over the last decade, SMC use has expanded to cover the most vulnerable group, i.e. children <5 y of age in high-transmission regions in sub-Saharan Africa. These well-established interventions are the core of malaria control programs; however, progress has plateaued despite efforts to improve their coverage and effectiveness. These interventions might therefore have reached a ‘saturation point’, with new interventions needed for further progress. The 2019 Lancet Commission on malaria eradication^62^ did not expect an effective vaccine to arrive until 2035 and considered this the primary barrier to eradication. Given the licensing of two malaria vaccines (in 2022 and 2023) and the promising development of mAbs and gene drives, there is an expanding range of tools for control and elimination of malaria. As these interventions are rolled out, understanding their feasibility and interactions (i.e. synergy) should be a priority. First, can multiple prevention measures use a single delivery framework to avoid replication of this cost, improving overall cost-effectiveness? This is essential as funding for global health is declining, meaning competition for resources becomes fiercer, leading to prioritisation of the most cost-effective interventions. Targeting areas with the greatest potential reduction in burden or those where elimination would save the most in future prevention costs that are no longer necessary are the most obvious candidates for this. Second, are the effect sizes of control measures indeed multiplicative?^63^ Encouragingly, Chandramohan et al.^36^ demonstrated the cumulative benefit of SMC and vaccination. Similar studies that investigate whether this is replicated by R21/Matrix-M and determine the most effective combinations of interventions will allow for integration into successful elimination programs. Rather than a one-size-fits-all approach, tailored solutions to the specific needs of communities and local transmission patterns that work within the constraints of available funding should be sought. Achieving sustained compliance with this ever-increasing raft of interventions may prove challenging, meaning health literacy and community engagement become increasingly vital.

## Conclusions

Population control strategies are in three broad phases: ITNs and SMC are focussed on consolidation of current success, R21/Matrix-M is moving towards availability at a similar scale as ITNs and SMC and emerging technologies such as mAbs and gene drives are making progress through trials. If these technologies can be combined successfully, they could contribute significantly towards the GTS goals for reductions in malaria mortality.^39^

## Data Availability

The data are available in the article and references.
